# Indications Derived from the Bed

**Published:** 1897-12

**Authors:** 


Indications Derived from the Bed: Not especially related to the foregoing paper of Dr. Morrow.

The patient fears to go to bed alone, Causticum. Bed sores, Calendula. Patient sits up in bed and throws himself upon it, Iodine, Hepar, Calcarea. Patient thinks some one is in the bed with him, Apis, Petroleum. It is characteristic of Petroleum.

